# Prevalence, related factors and maternal outcomes of primary postpartum haemorrhage in governmental hospitals in Kabul-Afghanistan

**DOI:** 10.1186/s12884-020-03123-3

**Published:** 2020-07-28

**Authors:** Shirin Shahbazi Sighaldeh, Adela Nazari, Raziyeh Maasoumi, Anoshirvan Kazemnejad, Ziba Mazari

**Affiliations:** 1 Reproductive health department, School of Nursing and Midwifery, Tehran University of Medical Sciences, P.O. Box 6459, Tehran, Iran; 2 Midwifery department, School of Nursing and Midwifery, Kabul Medical University, Kabul, Afghanistan; 3 Reproductive health department, School of Nursing and Midwifery, Tehran University of Medical Sciences, P.O. Box 6459, Tehran, Iran; 4 Department of biostatistics, Tarbiat Modares University, P.O. Box 8288, TMU, Tehran, Iran; 5 Midwifery department, School of Nursing and Midwifery, Kabul Medical University, Kabul, Afghanistan

**Keywords:** Observational design, Abnormal bleeding, Afghanistan women, Atony, Bleeding, Hemorrhage

## Abstract

**Background:**

To determine the prevalence, related factors and maternal outcomes of primary PPH in governmental hospitals in Kabul Afghanistan.

**Methods:**

An observational study was designed to determine the prevalence, related factors and maternal outcomes of primary PPH in governmental hospitals in Kabul-Afghanistan. The population of this study consisted of all women who gave birth to a child between August and October 2018. The structured checklist was used to collect the data from patients who were suffering from primary PPH.

**Results:**

Among the 8652 women who were observed, 215 (2.5%) of them suffered from primary PPH and 2 (0.9%) of them died under caesarean section. The most common related factors of primary PPH were uterine atonia (65.6%), previous PPH (34.9%), prolonged labor (27%), genital tract trauma (26.5%), and induction of labor (20.5%). The most common maternal outcomes of primary PPH were respiratory failure (7%), hysterectomy (6%), and hypovolaemic shock (5.1%).

**Conclusions:**

According to our findings, the major cause of postpartum bleeding was uterine atonia. Therefore, postpartum care of women is essential, especially for those with previous PPH and prolonged labor that require more attention.

## Background

A wealth of literature recognizes postpartum haemorrhage (PPH), a life-threatening event in a woman’s life due to extreme bleeding during and after the third stage of labor. Among the obstetrical complications, the most common one is PPH that affects up to 18% of deliveries. Globally, it is responsible for 35–55% of peripartum maternal deaths [1]. Traditionally, primary PPH is defined as blood loss exceeding 500 ml within 24 h after vaginal delivery, or blood loss exceeding 1000 ml following caesarean section (CS) [2]. Two-thirds of women who have PPH actually manifest no risk factor which makes it hard to predict PPH by risk factor [3].

Childbirth as a life threatening event for women represents one of the greatest inequities in global health. Every year 500,000 maternal deaths occur across the globe, 99% of which are in the low-income countries [4]. PPH with case fatality rate of 1% is one of the leading causes of maternal deaths that occur in over 10% of all births. In the developing world, 125 million children are born annually, in every 1000 births one mother dies due to PPH [1]. Specifically, every 7 min PPH takes the life of one women [5].Totally, sever haemorrhage accounts for 25 % of all maternal deaths [1].

Obstetric haemorrhage is a fatal event in a woman’s life that has its place at the top of the causes of maternal death and morbidity, especially in developing countries. In Africa and Asia, it is responsible for more than 30% of all maternal deaths [1]. In contrast, in developed countries such as the UK and the USA obstetric haemorrhage causes just 3.4% [6] and 11.4% [7] of maternal deaths, respectively. However, Afghanistan is among the countries which have the highest maternal deaths in the world. In this war-torn country, the ratio of maternal mortality is 400 per 100,000 live births [8]. Haemorrhage is the leading cause of maternal mortality in Afghanistan that accounts for 38% of all those deaths. To be more specific, 30% of maternal deaths are related to PPH [9]. Moreover, Afghanistan is also among the countries with the highest fertility rates in the world. In this low income country, the fertility rate is 5.1 children per woman [10]. It means this life threatening event occurs repeatedly in the life of Afghan women. High fertility rate and limited resources to provide obstetrical care for mothers make the situation more complicated for the health system [11].

Etiology of PPH is heterogeneous which has various well-known post-delivery causes such as retained placental tissue, genital tract trauma, with a major cause being uterine atonia [12]. Etiology of it is classified into four main groups including 1) uterine atonia, 2) placental problems including retained placenta and abnormal placental implantation, 3) genital tract trauma, and 4) systemic medical disorders (including inherited and acquired coagulation defects). The most common cause of PPH is uterine atonia that accounts for 80% of cases of primary PPH [13].

Some diseases have association with PPH including blood transfusion, renal failure, coagulation deficiencies and are also associated with long-term morbidities, including anemia, hysterectomy. Moreover, with other surgical procedures to reduce blood flow to the uterus and their subsequent consequences, infertility has to be considered as a consequence of PPH, although it is difficult to quantify their burden [12, 14, 15].

The most mournful result of PPH is maternal mortality that adversely impacts every family member, especially the child that is left behind [16].

Fortunately, there is still hope of preventing this plaintive event, a wealth of literature supports the claim that PPH can be prevented. Various approaches might be used taking the setting and availability of skilled birth attendants and supplies into consideration [17]. According to a metanalysis, prophylactic injection of tranexamic acid within 10 min after vaginal delivery will reduce the risk of primary PPH [18] and in women with established PPH the use of this medication can reduce the risk of adverse outcome like hysterectomy [19]. It has demostrated that early and aggressive treatment of PPH is a determinant of decreasing the morbidity and mortality associated with this worldwide health issue [20, 21].

The overall aim of this study was to determine the prevalence, related factors, and maternal outcomes of primary PPH. Determining the prevalence, related factors and maternal outcomes of primary PPH will provide the health care providers and policymakers (Ministry of Public Health) with the information of planning interventions to improve the care of women during pregnancy, labor and after delivery to reduce the impact of primary PPH and its outcomes.

## Methods

This study was conducted with observational design, to determine the prevalence, related factors and maternal outcomes of primary PPH. In this study, all the factors that could directly or indirectly be associated with massive bleeding were named as related factors.

The study recruitment was implemented in postpartum wards in four governmental hospitals, Malalai Zijenton Maternal Hospital, Rabia Blkhi Hospital, 102 beds of Khair Khana Hospital and Isteqlal Hospital, in Kabul, Afghanistan. All these hospitals have gynecology and maternity ward and neonatal intensive care unit (NICU). Malalai Zijenton Maternal Hospital has 220 beds and is the largest governmental gynecological and obstetrical hospital in Afghanistan. The hospital has 520 health providers and services personnel, 95% of them are women. Among them there are 88 midwives and 6 obstetricians, 3 attendings and 15 gynecology students that provide health services in the maternity ward. This hospital has an average of 2000 vaginal and cesarean section deliveries monthly, and has an average of 25,000 deliveries annually. Rabia Balkhi Hospital is the only women’s complex hospital in Afghanistan with 270 beds. The hospital has 431 health providers and services personnel in various departments and 95% of them are women. Among them there are 85 midwives and 6 obstetricians that provide health services in the maternity ward. The hospital has an average of 2000 vaginal and cesarean section deliveries monthly and has an average of 24,000 deliveries annually. Khair Khana Hospital has 102 beds and 350 health providers and services personnel in various departments. Among them there are 75 midwives and 6 obstetricians that provide health services in the maternity ward. The hospital has an average of 1000 vaginal and cesarean section deliveries monthly and has an average of 12,000 deliveries annually. Istiqlal hospital has 335 health providers and services personnel in various departments. Among them there are 70 midwives and 3 obstetricians, 3 attendings and 24 gynecology students that provide health services in the maternity ward. It has an average of 1000 vaginal and cesarean section deliveries monthly and has an average of 12,000 deliveries annually. During the last 3 years, the mentioned hospitals have had about 75,000 births per year, which the highest birth rate was nested into Malalai Zijenton Hospital and the lowest birth rate was nested into the 102 bed Khair Khana hospital, 26,500 deliveries per year and 13,439 deliveries per year, respectively. The number of cesarean sections in these Hospitals accounts for 15 to 20% of all deliveries. Isteqlal Hospital, 102 beds of Khair Khana Hospital, Rabia Blkhi Hospital and Malalai Zijenton Maternal Hospital have an average of 203, 148, 295 and 362 cesarean sections per month, respectively.

The population of this study consisted of all patients who delivered between August 2018 and October 2018, during the study period, and they were hospitalized in mentioned hospitals at the time of the study. The inclusion criteria included women who delivered in these four governmental hospitals or at home, and had primary PPH after normal vaginal delivery or cesarean section, within 24 h after birth. Women who were referred from other maternal health facilities or women who gave birth at home and then came to the hospital due to PPH could also participate in the study.

The sample size was calculated using the formula for a single population proportion. Based on a previous study from India, proportion of PPH is 0.9% (*P* = 0.009) [22]. At absolute precision of 0.002 (d = 0.002), level of confidence was 95% and included 1% of effusion cases.

$$ {\displaystyle \begin{array}{c}n=\frac{{z^2}_{\left(1-\frac{\alpha }{2}\right)}P\left(1-P\right)}{d^2}\\ {}=(1.96){}^2.(0.009).\left(1-0.009\right)/(0.002){}^2=8565.8\\ {}=8566+8566(0.01)=8652\end{array}} $$

The sampling method that was used in this study was convenience sampling technique. Researcher went to the hospitals in different shifts considering inclusion criteria to find eligible women. Of the 8652 women who gave birth in the four hospitals, 215 were eligible, all of them participated in the study and a checklist was completed for them. The checklist was designed by researchers and was developed by reviewing the scientific literature. It consisted of four sections: Section A included 8 questions about demographic characteristics, i.e. mother’s age, mother’s education, mother’s job, husband’s age, husband’s education, husband’s job, economic status and residential area. Section B included 19 questions about pregnancy and obstetric history, Section C included 24 questions about related factors of primary PPH, and section D included 9 questions about maternal outcomes of primary PPH. To determine the validity of the checklist that refers to the degree to which an instrument measures what is supposed to be measured [23], quality content validity was carried out based on the opinions of 10 faculty members at the School of Nursing and Midwifery, Tehran University of Medical Sciences. During validation suggestions regarding rearrangements, modifications and change in total number of questions were taken. These recommendations and the suggestions of experts were incorporated in the checklist and the instrument was finalized for the study. The checklist is included as a supplementary file (Additional file 1).

Data were collected between August 2018 and October 2018 by midwives who were trained to do this in postpartum wards. These collectors were the supervisors of caesarean section and vaginal delivery wards. They started collecting data from the first day of August from three governmental hospitals but the data from Isteglal hospital was collected by the researcher herself.

Information was obtained from hospital registers and caregivers, i.e. nurses, midwives or gynecologists and also through the interview with the women or their relatives. All collected information was double checked by researchers from date of entry, document number, date of discharge, and vital status of the women at the discharge date. Primary PPH was clinically assessed by the caregivers according to the direct complaint of the patient, examination of the patient’s signs and symptoms and observation of the patient’s bleeding. Also, the amount of PPH (less than or more than 1000^cc^) was estimated visually by caregivers. Routinely, at these hospitals, large diapers are used for all women after childbirth. They visually estimate the amount of bleeding based on the amount of blood that falls on the diaper. If a diaper is soaked in blood, the bleeding rate is estimated at 500 cc or more. If in a woman two or more diapers are stained with blood or if a woman has severe bleeding symptoms such as low blood pressure, rapid or weak pulse, or respiratory distress, the bleeding rate is estimated to be 1000^cc^ or more. Another reason for using adult diapers is that due to the high number of deliveries, sometimes two women are receiving care on the same bed, so adult diapers are used to prevent bed contamination.

The researcher has controlled the data collecting by messaging and calling the supervisors via mobile every day and getting information about the number of checklists that were filled out from each included woman and the number of vaginal births and caesarean section births. The number of primary PPH was between 2 and 7 cases per day. Every 2 weeks, the researcher referred to governmental hospitals to get the checklists from the supervisors. Totally, collecting and double checking of checklists by the researcher took 2 months and 20 days.

Written informed consent was obtained from all women participating in the study. The consent form is included as a supplementary file (Additional file 2). Besides, before getting data from hospital registers and caregiver’s information, the approval was obtained from the hospitals’ authorities. Also, obtaining permission from Ministry of Public Health of Afghanistan was done by collaboration with Kabul Medical University.

Data was analyzed by using the Statistical Package for Social Sciences version 16. The frequency and percentage were used to report the socio-demographic variable and mean and standard deviation were used to report the proportion of primary PPH occurrence, related factors and outcomes of primary PPH.

## Results

From the source population of 8652 deliveries, we identified 215 women with primary PPH of > = 500 cc or > =1000 cc, giving a prevalence of 0.025 (2.5%). Among 215 cases of primary PPH, illiterate women were more than others (62.8%). Majority of them were housewives (96.7%). In addition, one third of the husbands had primary and secondary school degree (33.5%). Also, a large number of them were self-employed (49.3%) (Table 1). The mean age of cases was 27.15 ± 6.3. The youngest woman was 14 years old and the oldest was 50 years old (Table 2). More than half of women with PPH had had their previous delivery at hospital (58.60%). Almost all of the women had their current delivery in hospital, 214 (99.5%). Only one woman (0.5%) had given birth at home. Half of the women had 2 to 3 children (47.4%). One-third of the women have had prior PPH (34.9%). Three-fourths of the women had prenatal care in their current pregnancy (74.4%). More than half of the women had regular prenatal care in their current pregnancy (53%). Majority of women had vaginal delivery in their current delivery (57.7%). Three-fourths of women had uterine bleeding (74.9%). In terms of the origin of bleeding 15.3% of women had vaginal bleeding, 5.6% of women had cervix bleeding and the rest of them had perineal bleeding (4.2%). It was estimated that in the majority of women (54.4%) the amount of bleeding was between 500 and 999 cc while 98 women (45.6%) had bleeding equal to or more than 1000 cc. Majority of women (54.9%), had been diagnosed by signs of primary PPH while 45.1% were diagnosed by direct patient’s complaint from abnormal bleeding. A large number of women had been primarily diagnosed by visual estimation of midwives (73%), and 24.2% of women had been primarily diagnosed by visual estimation of obstetricians (Table 3).

**Table 1:** Demographic characteristics

Demographic variables	Frequency N (%)
Mother’s education	
Illiterate	135 (62.8)
Primary and Secondary	65 (30.2)
School	12 (5.6)
High School	2 (0.9)
Diploma	1 (0.5)
Higher Education	
Mother’s job	
Housewife	208 (96.7)
Employed	7 (3.3)
Husband’s education	
Illiterate	71 (33)
Primary and Secondary	72 (33.5)
School	49 (22.8)
High School	19 (8.8)
Diploma	4 (1.9)
Higher Education	
Husband’s job	
Employed	3 (1.4)
worker	81 (37.7)
retired	3 (1.4)
free	106 (49.3)
jobless	22 (10.2)
Economic status	
Optimal	18 (8.4)
Medium	162 (75.3)
Undesirable	35 (16.3)
Residential area	
Urban	125 (58.1)
Suburb	80 (37.2)
Rural	10 (4.7)

**Table 2:** Mother’s age

Variables	mean	SD	min	max
Mother’s age	27.15	6.3	14	50

**Table 3:** Pregnancy and obstetric characteristics

Pregnancy and obstetric variables	Frequency N (%)
Place of Previous Delivery	
home	89 (41.40)
hospital	126 (58.60)
Place of Current Delivery	
home	1 (0.5)
hospital	214 (99.5)
Number of Parity	
Nulliparous	39 (18.1)
2–3	102 (47.4)
4–5	41 (19.1)
> 5	33 (15.3)
Gestation	
Singleton	208 (96.7)
Multiple	7 (3.3)
Abortion	
Yes	38 (17.7)
No	177 (82.3)
Number of Abortion	
1	29 (13.5)
2	7 (3.3)
3	2 (0.9)
Stillbirth	
Yes	40 (18.6)
No	175 (81.4)
Number of Stillbirths	
1	33 (15.3)
2	7 (3.3)
Previous Type of Delivery	
Vaginal	128 (73.6)
Cesarean section	6 (3.4)
Instrumental	6 (3.4)
Spontaneous	34 (19.5)
Previous large baby (babies weighing more than 4000 g)	
Yes	8 (3.7)
No	207 (96.3)
Previous PPH	
Yes	75 (34.9)
No	140 (65.1)
Having prenatal care in the current pregnancy	
Yes	160 (74.4)
No	55 (25.6)
Having regular prenatal care	
Yes	114 (53)
No	101 (47)
Type of current delivery	
Vaginal	124 (57.7)
Cesarean section	63 (29.3)
Instrumental	19 (8.8)
Spontaneous	9 (4.2)
Anatomical location of the bleeding	
Uterus	161 (74.9)
Vagina	33 (15.3)
Perinea	9 (4.2)
Cervix	12 (5.6)
Estimated amount of bleeding (cc)	
500_999	117 (54.4)
≥ 1000	98 (45.6)
How the primary PPH was diagnosed	
sign and symptoms	118 (54.9)
patient’s complaint	97 (45.1)
Primarily who diagnosed the primary PPH	
Nurse	1 (0.5)
Midwife	157 (73)
Obstetrician	52 (24.2)
Others^a^	5 (2.3)
Final confirmation person	
Midwife	54 (25.1)
Obstetrician	146 (67.9)
Others^a^	15 (7)

^a^Others are residents and MD doctors

Neonate mean weight was 3200.5 ± 525.9. Maximum weight of neonate was 4500 g and minimum weight of neonate was 1200 g (Table 4).

**Table 4:** Neonate weight

Variable	mean	SD	min	max
Neonate weight	3200.47	525.9	1200	4500

Most common related factors of primary PPH were uterine atonia (65.6%), followed by previous PPH (34.9%) and prolonged labor (27%). Others were genital tract trauma (26.5%), induction of labor (20.5%), precipitate labor (18.1%), and placenta retention (16.7%) (Table 5). In 44 women, the bleeding had adverse outcomes such as respiratory failure in 7%, hysterectomy in 6%, hypovolemic shock in 5.1%, and mortality in 0.9%. Both women who died had prolonged labor and death occurred under cesarean section after a massive bleeding (Table 6).

**Table 5:** Correlates of primary postpartum hemorrhage

Correlates of primary PPH variables	Frequency N (%)
Uterine atony	141 (65.6)
Previous PPH	75 (34.9)
Prolonged labor	58 (27)
Genital tract trauma	57 (26.5)
Induction of labor	44 (20.5)
Precipitate labor	39 (18.1)
Placenta retention	36 (16.7)
Anemia	33 (15.3)
Multi parity	23 (10.7)
Hypertensive disorder	12 (5.6)
Premature rupture of membrane (PROM)	11(5.1)
Uterine rupture	9 (4.2)
Chorioamnionitis	8 (3.7)
Multiple pregnancies	7(3.3)
Hematoma	7 (3.3)
Abruption	6 (2.8)
Diabetes mellitus	2 (0.9)
Poly hydroaminus	2(0.9)
Magnesium sulfate	2 (0.9)
Unidentified correlate	2 (0.9)
Shoulder dystocia	1 (0.5)

**Table 6:** Maternal outcomes of primary postpartum hemorrhage

Maternal outcomes of primary PPH variables	Frequency N (%)
Respiratory failure	15 (7)
Hysterectomy	13 (6)
Hypovolemic shock	11 (5.1)
Internal iliac ligation	7 (3.3)
Renal failure	6 (2.8)
Heart failure	3 (1.4)
Death	2 (0.9)
none of them	171 (79.5)

## Discussion

In this study the prevalence of primary PPH was 2.5%. The diagnosis and judgment about the PPH may not be correct by caregivers and they might have estimated the bleeding of less than 500 cc as PPH. According to other studies, the prevalence of PPH has a wide regional variation ranging from 7.2% in Oceana to 25.7% in Africa and also around 8% of women suffer from PPH in Latin America and Asia with a prevalence of about 13% in Europe and North America. Additionally, Africa has a high number of sever PPH (5.1%), followed by a prevalence around 4.3% in North America with the lowest prevalence in Asia (1.9%) [24]. In our study the prevalence of primary PPH may be due to the specific geography and residence culture which was comparable to Edhi et al. [25] and Lotfalizade et al’s [26] studies from Pakistan and Iran where they found a prevalence of 1.74 and 1.17%, respectively.

Kodla [22] reported the prevalence of sever PPH around 0.9% based on the definition of sever PPH > =1500 CC which is not similar to our own.

Age above 25 years significantly increased hemorrhage and emphasizes the importance of not getting pregnant until at an older age [27]. The high incidence of bleeding contributed to age may be due to rising parity, uterine atonia, complicated placenta and increasing cesarean section rates. In our study, the age range of the women was between 14 and 50 with a mean age of 27.15 ± 6.3 years. Similar findings were found by Edhi et al. [25] and Lotfalizade et al. [26]. They reported mean age as 26.153 ± 7.37 from Pakistan and 28 years ranging from 18 to 42 years from Iran.

A large number of study participants were illiterate women (62.8%) and majority of them were housewives (96.7%), as well the majority of women’s husbands were primary and secondary school level (33.5%) and most of them were not employed (49.3%). It demonstrates the worse thing about the status of the women that suffered from primary PPH in governmental hospitals in Kabul and supports the finding of The National Reproductive Health Strategy 2006–2009 which shows that a large percentage of families in Afghanistan (> 70%) are facing limitations, especially those seeking help because of the expenditures or insignificant quality provided by the accessible health care center [28]. The costs of obstetrical care can be an important barrier to the low level income people [11].

A large proportion of women experienced their previous and current delivery in hospital, (71.6%) and (99.5%), respectively. As opposed to Edhi et al. [25], we found a large number of multiparous (2–3 parity) women in our findings (47.4%), similar to Kodla’s [22] findings. Additionally, a smaller percentage of our findings was multiple gestations (3.3), which was comparable to Kodla’s [22] findings.

Almost half of our participants had vaginal delivery in their history and current delivery, (73.6%) and (57.7%), respectively. As opposed to Edhi et al. [25] who reported that the most common type of delivery was spontaneous vaginal delivery (61.5%), and Kodla [22], which reported that cesarean section delivery was the most common type of delivery in their study (55.65%).

Our findings showed three-quarters of women attended prenatal care units and one fourth of them did not get checked up during pregnancy, (74.4%) and (25.6%), respectively. Also, around 53% of women had visited prenatal care facilities regularly. National guidelines recommend having prenatal care a minimum of 4 times [29]. Our findings supported Rahmani et al’s [30] results, the lack of knowledge among women and their families regarding the importance of prenatal care, besides financial reasons, lack of obstetric care by a female doctor, public transportation problems, women’s dissatisfaction with the attitudes and behavior of health provider, low income level and insignificant quality of care, contributed to this issue.

The most common related factor of primary PPH in this study was uterine atonia which contributed to 65.6% which is comparable to Lotfalizade et al. [26] (55.6%), Neflot et al. [31] (60.4%) and Yinkaoyelese et al. [32] (70%), respectively and was more than Kodla’s [22] study (33.9%).

In our findings prior PPH was around 34.9% and according to Neflot et al. [31] it was the strongest related factors of sever PPH (adjusted OR = 8.97, 95% CI: 5.25–15.33).

In our study the contribution of prolonged labor and genital tract trauma in PPH were similar, 27 and 26.5%, respectively. Induction of labor (20.5%), precipitate labor (18.1%), placenta retention (16.7%), anemia (15.3%), and multi parity (10.7%) were other important contributors. In the study of Edhi et al. [25], uterine atonia was reported in 6 (*P* = 0.68) and cervical or vaginal tear in 2 cases (*P* = 0.336). The related factors of PPH are different in previous study based on their definitions.

According to our findings, respiratory failure (6%), hysterectomy (5.1%) and hypovolemic shock, (3.3%) were the most common maternal outcomes of primary PPH. Maternal mortality occurred in 2 cases (0.9%). Both of these women had prolonged labor and death occurred under emergent cesarean section after a massive bleeding. Edhi et al. [25] reported that repair of cervical and vaginal tear were in 6 (*P* = 0.677), internal iliac ligation in 2 (*P* = 0.336), replacement of uterine inversion in 2 (*P* = 0.505) and contrary to our results, no maternal deaths were recorded in Edhi et al. [25] and Lotfalizade et al. [26]. Olowokere et al. [33] found renal failure 2.9%, heart failure 5.9%, hypovlaemic shock 14.7, and 1.5% of cases were maternal death in the tertiary health care facilities. Kodla [22] found cases of sepsis 8.95%, acute renal failure 8.52, 10.31% of cases underwent hysterectomy, 8.52% accounted for multi-organ failure, 4.93% of cases accounted for ARDS and maternal death occurred in 21.73% of cases.

Contrary to existing studies, our study reported that the rate of respiratory failure was more than hemorrhagic shock. We supposed that this issue may be rooted in the context of Afghanistan’s hospitals. Since in Afghanistan vaginal delivery is routinely controlling by midwives, for some cases of PPH they may not have access to a gynecologist immediately and therefore, they rush to the start of serum therapy. This can lead to overloading of the lungs and result in the symptoms similar to those of respiratory failure. On the other hand, due to the fact that at the time of this study, there was no protocol for bleeding management in Afghanistan, it is possible that a mild shock was not detected by the midwifes or that the mother’s respiratory efforts were reported as respiratory failure.

### Strengths and limitations of the study

The main strength of current study was our data collectors who were the supervisors of the vaginal and cesarean section delivery wards. Thus, it was expected that all cases of primary postpartum hemorrhage would be recorded by them. Another strength of current study was double checking of all data by researcher. Last but not least, the study was conducted in maternity wards in all government hospitals. Therefore, the results can be generalized to all government hospitals or provide important insights for hospitals with similar characteristics.

The low educational level of the participants could be a limitation, so data collectors explained all the questions to the participants and checklists were completed through interview.

First and foremost, there is a possibility that some cases of PPH were misclassified, ignored or underreported because blood loss is routinely estimated visually in government hospitals of Afghanistan, and there are no standard methods for quantifying the amount of bleeding. However, we asked caregivers to give us further information of potential cases of PPH by reporting blood transfusion cases or other therapeutic measures. Regarding the verbal recommendation of Ministry of Public Health, the length of hospitalization is 6 h after vaginal delivery in governmental hospitals. Thus, most participants of current study were discharged from hospitals soon and we had no information about the fate of them after discharge.

Another limitation is that other related factors may be involved in the development of primary PPH that have not been analyzed in this study.

Since in this study data had to be collected from four hospitals at the same time, the researcher alone could not do that. Because of this, data were collected by different collectors. Thus, another limitation of our study was the quality of the data that might vary due to the different people who collected the data. The researcher tried to monitor the data collection process and recheck the information of some questionnaires randomly to make sure that they had gathered the data correctly.

Also, due to the large number of deliveries, it was not possible to collect data from healthy women to compare their conditions to the participants.

## Conclusion

The prevalence of primary PPH was 2.5%. Among demographic characteristics of women with primary PPH, educational level, being housewife and self-employment of women’s husbands were the most common related factors of primary PPH. Among pregnancy and obstetrics characteristics, multiparity, uterine atonia, previous PPH, prolonged labor and genital tract trauma were the most common related factors of primary PPH. Among women with primary PPH, the most common maternal outcomes were respiratory failure, hysterectomy, and hypovolemic shock.

Since women in postpartum do not have more than 6 h of hospitalization in Afghanistan, there is no comprehensive assessment of bleeding for the first 24 h after delivery. Therefore, it is recommended that the duration of post-partum hospitalization of women be extended longer than the current recommendation of 6 h, and a follow-up system established to monitor women after discharge for at least 24 h. Symptoms of abnormal postpartum bleeding should be taught to women and their family. In addition, it is recommended that a standardized method for quantification of the blood loss be used in Afghanistan hospitals. Providing in-service training for all midwives should be done to diagnose, inform, manage, and prevent PPH. Also, it is suggested that other related factors that may be involved in the development of primary PPH be addressed in further studies such as duration of labour stages, prophylactic use of ecbolics, management of 3rd stage of labour, pre-existing medical conditions, etc.

## Supplementary information

**Additional file 1.** Study’s checklist. The structured checklist was used to collect the data from all patients. The checklist consists of four sections: Section A for demographic characteristics including 8 questions; Section B for pregnancy and obstetric characteristics including 19 questions; Section C for correlates of primary PPH including 24 questions; Section D for maternal outcomes of primary PPH including 9 questions.

**Additional file 2.** Study’s consent form. Written informed consent was obtained from all participants.

## Data Availability

Contact Adela Nazari; email: adela.nazari@yahoo.com to request access to data and materials.

## Abbreviations

PPH: Primary Postpartum Haemorrhage
CS: Caesarean Section
USA: United States
UK: United Kingdom
NICU: Neonatal Intensive Care Unit
TUMS: Tehran University of Medical Sciences
OR: Odds Ratio
CI: Confidence Interval
ARDS: Acute Respiratory Distress Syndrome
