# Supplementary material for: Prevalence, related factors and maternal outcomes of primary postpartum haemorrhage in governmental hospitals in Kabul-Afghanistan
Source: BMC Pregnancy Childbirth. 2020 Jul 28;20:428. doi: 10.1186/s12884-020-03123-3 (PMC7390104; doi:10.1186/s12884-020-03123-3)
Supplement: Supplementary file 1 — Additional file 1. Study’s checklist. The structured checklist was used to collect the data from all patients. The checklist consists of four sections: Section A for demographic characteristics including 8 questions; Section B for pregnancy and obstetric characteristics including 19 questions; Section C for correlates of primary PPH including 24 questions; Section D for maternal outcomes of primary PPH including 9 questions. [file 12884_2020_3123_MOESM1_ESM.docx]

**Supplementary file**

**Checklist of primary postpartum haemorrhage**

**The applicant participates in a research project, the participation is voluntary, and all information will remain confidential.**

**Document No: Date and time: Hospital** name:

**Section A: demographic characteristics:**

1. **Mother’s age: …………… year**
2. **Mother’s education:**

1- **Illiterate**: 🞏 2- Primary and Secondary School: 🞏 3- High School: 🞏

4- Diploma: 🞏 5- Higher Education: 🞏

1. **Mother’s job**: 1- Housewife: 🞏 2- Employed: 🞏
2. **Husband’s education**:

1- Illiterate: 🞏 2- Primary and Secondary School: 🞏 3- High School: 🞏

4- Diploma: 🞏 5- Higher Education: 🞏

1. **Husband’s job:**

1- Employed: 🞏 2- worker: 🞏 3- retired: 🞏 4- free: 🞏 5- jobless: 🞏

1. **Economic status:** 1- Optimal: 🞏 2- Medium: 🞏 3- undesirable: 🞏
2. **Residential area:** 1- Urban: 🞏 2- subUrb: 🞏 3- Rural: 🞏

**Section B: Pregnancy and obstetric characteristics:**

1. **Place of previous delivery:**

1- Home: 🞏 2- hospital: 🞏 3- on the way of hospital: 🞏

1. **Place of current delivery:**

1- Home:🞏 2- hospital: 🞏 3- on the way of hospital: 🞏

1. **Number of Parity:**

1- Nulliparous: 🞏 2- 2-3 parity: 🞏 3- 4-5 parity: 🞏 4- more than 5 parity: 🞏

1. **Gestation**: 1- Singleton: 🞏 2- multiple: 🞏
2. **Abortion**: 1- yes: 🞏 2- No: 🞏

if yes, number of abortion…………………..

1. **Stillbirth**: 1- yes: 🞏 2- No: 🞏

if yes, number of stillbirth………………..

1. **Previous type of delivery**: 1- Vaginal delivery: 🞏 2- cesarean section delivery: 🞏 3- instrumental delivery: 🞏 4- elective cesarean: 🞏 5- spontaneous delivery: 🞏
2. **Previous large baby**: 1- yes: 🞏 2- No: 🞏
3. **Previous placenta accreta or percreta**: 1- yes: 🞏 2- No: 🞏
4. **Previous PPH**: 1- yes: 🞏 2- No: 🞏
5. **Having prenatal care in current pregnancy :** 1- yes: 🞏 2- No: 🞏
6. **Having regular prenatal care:**  1- yes: 🞏 2- No: 🞏
7. **Type of current delivery:** 1- Vaginal delivery: 🞏 2- cesarean section delivery: 🞏 3- instrumental delivery: 🞏 4- elective cesarean: 🞏 5- spontaneous delivery: 🞏
8. **Fetal or neonate weight**: ……………………gr
9. **Anatomical location of bleeding:**

1- Uterus: 🞏 2- vagina: 🞏 3- perinea: 🞏 4- cervix: 🞏

1. **estimated the amount of bleeding:** 1- ≥500 cc: 🞏 2- ≥1000 cc: 🞏
2. **how to diagnosed the primary PPH:**

1- by singe and symptoms: 🞏 2- by patient’s complaint from PPH: 🞏

1. **Primarily who diagnosed the primary PPH?**

1- Nurse: 🞏 2- midwife: 🞏 3- obstetrician: 🞏 4- others: 🞏

1. **Final confirmation person:**

1- Nurse: 🞏 2- midwife: 🞏 3- obstetrician: 🞏 4- others: 🞏

**Section C: correlates of primary PPH**

**correlates of primary PPH:**

1- Multiple pregnancy: 🞏 2- Uterine atony: 🞏 3- Previous PPH: 🞏 4- Placenta previa: 🞏 5- Genital tract trauma: 🞏 6- Abruption: 🞏 7- Coagulopathy: 🞏 8- Uterine rupture: 🞏 9- Hypertensive disorder: 🞏 10- Diabetes mellitus: 🞏 11- premature rupture of membrane (PROM): 🞏 12- Induction of labor: 🞏 13- Shoulder dystocia: 🞏 14- Anemia: 🞏 15 -precipitate labor: 🞏 16-prolonged labor: 🞏 17- poly hydroaminus: 🞏 18- multi parity: 🞏 19- placenta retention: 🞏 20- chorioamnionits: 🞏 21- magnesium sulfate: 🞏 22- uterine inversion: 🞏 23- hematoma: 🞏 24- unidentified correlate: 🞏

**Section D: maternal outcomes of primary PPH:**

1. **therapeutic outcomes (maternal outcomes):**

1- Renal failure: 🞏 2- Heart failure: 🞏 3- Hypovolaemic shock: 🞏 4- Respiratory failure: 🞏 5- Internal iliac ligation: 🞏 6- Hysterectomy: 🞏 7- Death: 🞏 8- none of them: 🞏

**Best regards**
