# Supplementary material for: Prevalence, related factors and maternal outcomes of primary postpartum haemorrhage in governmental hospitals in Kabul-Afghanistan
Source: BMC Pregnancy Childbirth. 2020 Jul 28;20:428. doi: 10.1186/s12884-020-03123-3 (PMC7390104; doi:10.1186/s12884-020-03123-3)
Supplement: Supplementary file 2 — Additional file 2. Study’s consent form. Written informed consent was obtained from all participants. [file 12884_2020_3123_MOESM2_ESM.docx]

**Supplementary file**

**(Information for participants) form**

**After the researcher introduces him/herself;**

“Prevalence, correlates, and maternal outcomes of primary postpartum hemorrhage in governmental hospitals in Kabul- Afghanistan in 2018- 2019.

”

- The aim of the research: To determine the prevalence, correlates and maternal outcomes of primary postpartum hemorrhage in governmental hospitals in Kabul- Afghanistan in 2018- 2019.
- I; (Adela Nazari) am the researcher and a student of higher education (master degree) in the department of maternal and child health at midwifery, medical university of Tehran, Iran. Sincerely doctor (Shirin Shahbazi Sighaldeh) is my research supervisor.
- Dear participant: due to agreement to participate in this research, you will be interviewed for your current state and a scientific assessment of of for this interview we will be in need of (20-30) mints of your time, and you will be interviewed only one time.
- Participant’s rights: participants have the right to step back from participation at any time or any step of the research.
- If wanted, the participant can have the chance to see the whole results of this research, after it has been finished. Participants have the right to ask for clarification about any of the interview, if it wasn’t already clear to them.
- Benefits and harms from participations:
- Participation in this research is for volunteer, participants will not be provided with any financial compensation for it, at the same time no payment will be received from the participations.
- Secrecy and confidentiality:
- In no way the participant’s name will be asked for, nor any personal or specific question, that may annoy the participant or she doesn’t like to answer it. We will make participants assure of the fact that their information will be kept safe, and apart from researcher, no other persons or parties will have access to them, and they won’t be used for any purpose other than research.

Feel free to share any opinions or suggestions with the researcher whenever and whenever you are, through the contacts below:

Email: [adela.nazari@yahoo.com](mailto:adela.nazari@yahoo.com)

Contact No, Afghanistan: 0785461442

Iran: 00989017602314

**(Informed consent) form**

Research title: “Prevalence, correlates, and maternal outcomes of primary postpartum hemorrhage in governmental hospitals in Kabul- Afghanistan in 2018- 2019.”

**Researcher’s name: Adela Nazari**

Dear participant: we would first like to thank you for agreeing to participate in this research, wish you a good health.

Dear participant: after signing this form, you are ready to be interviewed on all the steps and the parts of this research.

You as a participant should have all your information true, that you are going to share with the researcher (note that the information will be used for research purposes solely)

This form is proves that your participation is for volunteer, and you have the right to step back at any time from participation in the research.

Participant’s signature:

Researcher’s signature:

Date: / /2018-2019
